# The C-terminal peptide of CCL21 drastically augments CCL21 activity through the dendritic cell lymph node homing receptor CCR7 by interaction with the receptor N-terminus

**DOI:** 10.1007/s00018-021-03930-7

**Published:** 2021-09-29

**Authors:** Astrid Sissel Jørgensen, Emma Probst Brandum, Jeppe Malthe Mikkelsen, Klaudia A. Orfin, Ditte Rahbæk Boilesen, Kristoffer Lihme Egerod, Natasha A. Moussouras, Frederik Vilhardt, Pawel Kalinski, Per Basse, Yen-Hsi Chen, Zhang Yang, Michael B. Dwinell, Brian F. Volkman, Christopher T. Veldkamp, Peter Johannes Holst, Katharina Lahl, Christoffer Knak Goth, Mette Marie Rosenkilde, Gertrud Malene Hjortø

**Affiliations:** 1 Department of Biomedical Sciences, Faculty of Health and Medical Sciences, University of Copenhagen, Blegdamsvej 3B, Room 18.5.32., 2200 Copenhagen, Denmark; 2 Department of Immunology and Microbiology, University of Copenhagen, Copenhagen, Denmark; 3 Novo Nordisk Foundation, Center for Basic Metabolic Research, University of Copenhagen, Copenhagen, Denmark; 4 Department of Microbiology and Immunology, Medical College of Wisconsin, Milwaukee, WI USA; 5 Department of Cellular and Molecular Medicine, University of Copenhagen, Copenhagen, Denmark; 6 Department of Medicine, Roswell Park Comprehensive Cancer Center, Buffalo, NY USA; 7 Copenhagen Center for Glycomics, University of Copenhagen, Copenhagen, Denmark; 8 Department of Biochemistry, Medical College of Wisconsin, Milwaukee, WI USA; 9 Department of Chemistry, University of Wisconsin-Whitewater, Whitewater, WI USA; 10 Immunology Section, Lund University, 221 84 Lund, Sweden; 11 Section for Experimental and Translational Immunology, Department of Health Technology, Technical University of Denmark (DTU), Lyngby, Denmark

**Keywords:** Chemokine, CCR7, Glycosylation, Peptide, Dendritic cell

## Abstract

The endogenous chemokines CCL19 and CCL21 signal via their common receptor CCR7. CCL21 is the main lymph node homing chemokine, but a weak chemo-attractant compared to CCL19. Here we show that the 41-amino acid positively charged peptide, released through C-terminal cleavage of CCL21, C21TP, boosts the immune cell recruiting activity of CCL21 by up to 25-fold and the signaling activity via CCR7 by ~ 100-fold. Such boosting is unprecedented. Despite the presence of multiple basic glycosaminoglycan (GAG) binding motifs, C21TP boosting of CCL21 signaling does not involve interference with GAG mediated cell-surface retention. Instead, boosting is directly dependent on *O*-glycosylations in the CCR7 N-terminus. As dictated by the two-step binding model, the initial chemokine binding involves interaction of the chemokine fold with the receptor N-terminus, followed by insertion of the chemokine N-terminus deep into the receptor binding pocket. Our data suggest that apart from a role in initial chemokine binding, the receptor N-terminus also partakes in a gating mechanism, which could give rise to a reduced ligand activity, presumably through affecting the ligand positioning. Based on experiments that support a direct interaction of C21TP with the glycosylated CCR7 N-terminus, we propose that electrostatic interactions between the positively charged peptide and sialylated *O*-glycans in CCR7 N-terminus may create a more accessible version of the receptor and thus guide chemokine docking to generate a more favorable chemokine-receptor interaction, giving rise to the peptide boosting effect.

**Supplementary Information:**

The online version contains supplementary material available at 10.1007/s00018-021-03930-7.

## Introduction

Chemokine receptors belong to class A G-protein coupled receptors (GPCRs). They induce G_αi_ signaling and β-arrestin recruitment upon ligand binding leading to one of the most important downstream effects, control of leukocyte migration. A chemokine receptor may be targeted by multiple chemokines, and the same chemokine may act on several chemokine receptors giving rise to a highly promiscuous signaling network [1].

The activity of a receptor may be modulated to preferentially activate G_αi_ signaling, β-arrestin recruitment or other downstream effector pathways according to the ligand it interacts with or what tissue the receptor is expressed in giving rise to ligand and tissue bias respectively [1]. Biased signaling of the chemokines CCL19 and CCL21 at their common receptor CCR7 is a well-known phenomenon [2–4], yet the complete details governing the underlying mechanism behind this bias have yet to be revealed. These two chemokines interact differentially with the binding pocket of CCR7 resulting in different allosteric events and helical movements [4–6]. This opens unique possibilities for contact with downstream effector molecules like G_αi_ and β-arrestin giving rise to the observed bias. In general, CCL19 provides a stronger, more short-lived signal than CCL21, and CCL19 is efficient in both G_αi_ signaling and β-arrestin recruitment, whereas CCL21 is a weak stimulator of G_αi_ signaling and even weaker in β-arrestin recruitment, but at the same time elicits a prolonged signal from CCR7 through MAP-kinase activation in dendritic cells (DCs) [3, 4, 7].

Chemokine receptor activation is highly dependent on the interaction of the chemokine N-terminus with the receptor binding pocket leading to subsequent changes in receptor conformation [8]. As described by the (simplified) two-step, two-site activation model, the initial contact is mediated via chemokine core interactions with extracellular regions of the receptor, followed by the chemokine N-terminus docking into the transmembrane part of the receptor, leading to receptor activation. However, although CCL19 and CCL21 have different N-termini, the replacement of CCL19 N-terminus with CCL21 N-terminus did not affect the signaling activity of CCL19 at CCR7 [9, 10], implying that the different N-termini are not responsible for the lower overall potency of CCL21 compared to CCL19.

**Fig. 1:**
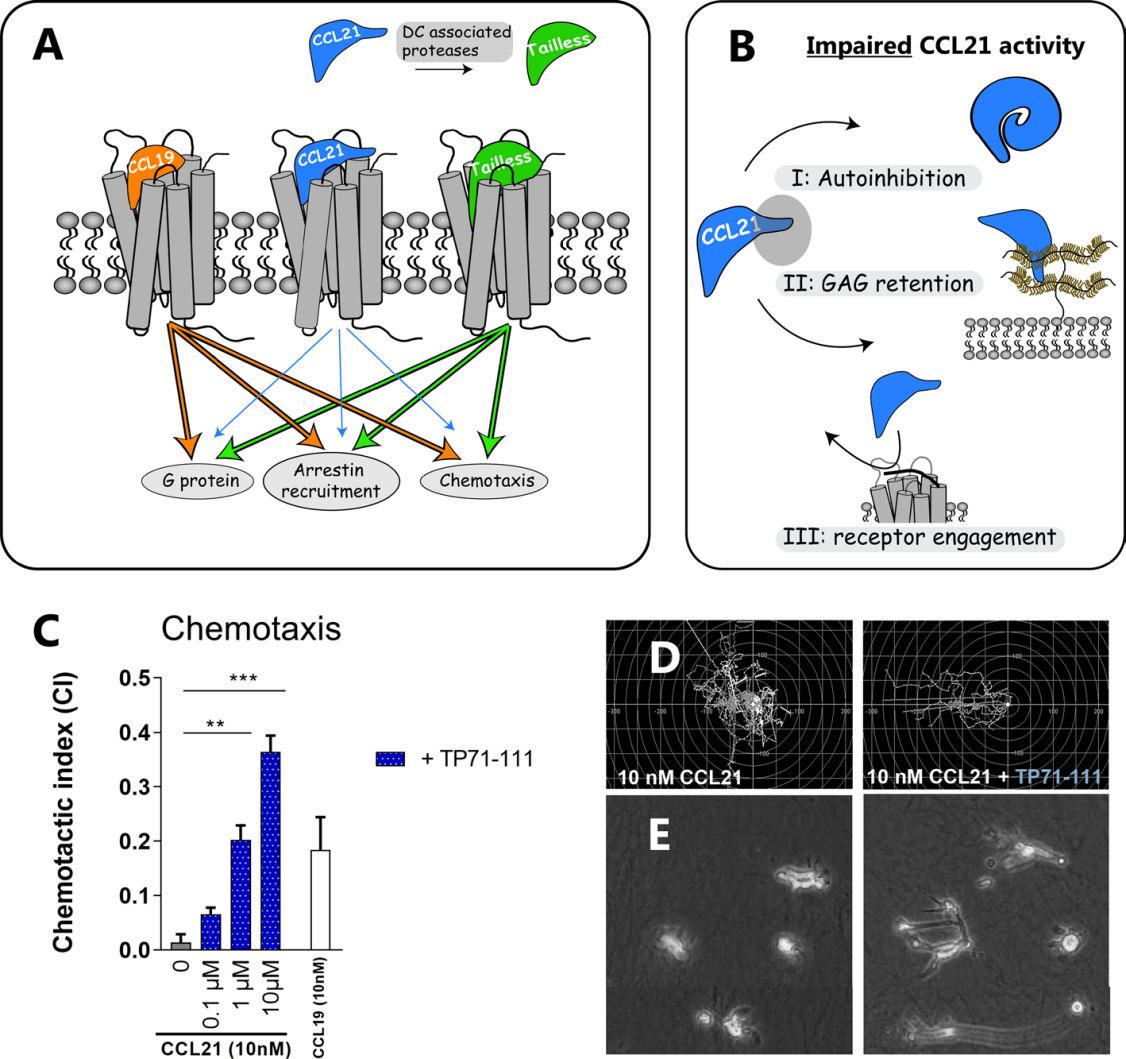
CCL21 C-terminal tail-peptide C21TP boosts CCL21 chemotaxis inducing potential in human primary DCs. **A** CCL19 and CCL21 induce biased signaling at their shared receptor CCR7, and CCL19 is a more potent ligand for G_αi_–signaling, β-arrestin recruitment and chemotaxis compared to CCL21. Removal of the C-terminal domain of CCL21 creates the more potent CCL21^Tailless^ that resembles CCL19 more than CCL21. **B** The impaired CCL21 activity is thought to reside within I) an auto-inhibitory function of its own C-terminus, II) chemokine GAG retention, or III) a different receptor engagement. **C** The effect of C21TP (TP71-111) on CCL21-induced chemotaxis was measured by time-lapse recordings of human moDCs naturally expressing CCR7. DC chemotaxis was measured in response to 10 nM CCL21. Blue bars show migration in the presence of C21TP (TP71-111) with the vertical numbers displaying the concentration of C21TP (0.1, 1 or 10 µM). Migration towards CCL21 only is shown as a grey bar. Migration towards 10 nM CCL19 alone shown to the right with a white bar. Statistical significances was determined using one-way ANOVA with Tukey’s multiple comparisons tests (*n* = 3–11). **P < 0.01, ***P < 0.001. **D** Spider diagrams depicting the DC migration pattern in response to 10 nM CCL21 alone or in the presence of 10 µM of C21TP (TP71-111). **E** Micrographs of the same migrating DCs demonstrating the morphological changes of DCs undergoing undirected (CCL21 only) versus directed chemotaxis (CCL21 in the presence of C21TP). Supplementary movies S1 and S2 illustrate the boosting effect exerted by C21TP on CCL21-induced DC chemotaxis

An important structural feature adding to the difference in receptor activation between CCL19 and CCL21 is the 37 amino acid extended positively charged C-terminus of CCL21, not mirrored in CCL19 [11, 12] (Fig. 1A). Previous studies suggest that CCL21 is auto-inhibited by its elongated C-terminus as it folds back upon the chemokine to enforce a conformation that is less active [13]. This so-called auto-inhibition model was suggested based on NMR studies revealing clear structural changes in CCL21 upon removal of the C-terminus generating a more potent ligand CCL21^Tailless^ [13]. Similar conformational changes and increases in signaling ability were observed in CCL21 in the presence of free polysialic acid leading to the hypothesis that interaction of CCL21 with polysialic acid adapts its structure to a conformation similar to that of CCL21^Tailless^ [13]. Mature DCs, one of the key immune cell types expressing CCR7, are responsible for the initiation of innate as well as adaptive immune responses. In DCs, CCR7 is polysialylated on both N- and *O*-glycans presumably, which seems to be important for CCL21 activation of CCR7 through a mechanism that causes unlocking of the auto-inhibited chemokine state in this cell-type [13, 14]. Both these types of glycosylation can act to modulate ligand receptor interactions. Thus in CCR5 sialic acid on *O*-glycans in the N-terminus are important for ligand binding [15] and in CCR7, N-glycosylation of the receptor N-terminus and extracellular loop 3 (ECL3) was shown to impose steric hindrance and thus negatively affect ligand-receptor interaction [14]. Similarly, sulfation of tyrosine residues in the N-terminus of many chemokine receptors affects ligand-receptor interaction, including CCR7, where the affinity of CCL21 is increased by tyrosine sulfation [16].

Glycosaminoglycans (GAGs) of the extracellular matrix (ECM) are important players in chemokine-induced receptor activation, with GAG retention of chemokines creating a reservoir on the cell surface [17]. GAG-bound chemokines can be freed through binding of other chemokines to the same GAGs, causing release of the less abundant chemokine to raise its effective concentration and thus allowing chemokine receptor interaction to occur. This theory is referred to as the chemokine cooperativity model [18]. Thus inherent high GAG affinity could lower the overall potency of a given chemokine [18].

We have recently published that although the GAG-binding C-terminus CCL21 may add to the observed biased signaling and differences in potency of CCL19 and CCL21, bias cannot be transferred by the C-terminus alone [19]. Hence the chimeric chemokine CCL19^CCL21−tail^ (CCL19 fused to the extended C-terminus of CCL21), although hampered in its ability to signal via G_αi_, does not display the same low potency in chemotaxis inducing potential as CCL21. In contrast to CCL19, CCL19^CCL21−tail^ binds extensively to DC surfaces, similar to what is observed for CCL21 [19]. Thus, the predominant theory that the low potency of CCL21 is mainly caused by C-terminus mediated retention of this chemokine on GAGs preventing fruitful interaction with CCR7 does not fully explain the difference in potencies observed between CCL19 and CCL21 [19].

CCL21^Tailless^, the C-terminally truncated CCL21, is a naturally occurring version of CCL21 generated upon proteolytic cleavage of the C-terminus by proteases released by activated DCs [20] and by plasmin [21], a protease involved in the regulation of blood clotting (Fig. 1A). This variant of CCL21 is much more potent as a chemotactic cue and could play an important role in potentiating lymph node (LN) homing of DCs during immune activation, with activated DCs hypothetically reinforcing the signal that causes them to relocate from the periphery to the LN during immune activation [4, 14, 20]. As CCL21 is expressed by lymphatic endothelial cells and is considered the major LN homing chemokine, such potentiation of CCL21 signaling is important to consider.

Thus, overall the positively charged, basic C-terminus of CCL21 negatively affects signaling and chemotaxis induced by CCL21 as quantified in multiple cell-lines as well as primary human DCs [19, 22] (Fig. 1A, B).

Interference with such internal antagonistic function of CCL21 could be a future means to boost immune activation in settings where this is required, e.g. in reactivation of the immune system against cancer. Here, in an attempt to outcompete the inhibitory action the basic C-terminus of CCL21 inflicts on the chemokine core domain, we use a molecular excess of the peptide that corresponds to the C-terminal part of CCL21, called C21TP, and investigate the effect of this peptide on signaling induced by CCL21. We find that the C21TP strongly potentiates the activity of CCL21. We look into the current models for impaired CCL21 activity (Fig. 1B) to investigate the mechanism of action by which the peptide boosts CCL21.

## Results

### CCL21 tail peptide greatly potentiates dendritic cell chemotaxis induced by CCL21

C-terminal truncation of CCL21 generates a more potent chemotactic chemokine, CCL21^Tailless^ (CCL21 1–79), an improvement thought to primarily be driven by a release from an auto-inhibited state otherwise enforced by the attached basic tail [13] and possibly also through the lower GAG affinity of CCL21^Tailless^. Here we set out to test if an excess of free CCL21 C-terminal peptide, C21TP (called TP71-111, CCL21 71–111), affects full-length CCL21 (residues 1–111) chemotaxis. Consistent with previous data, CCL21 is a very weak chemotactic cue at a concentration of 10 nM, whereas CCL19 composes a strong migratory signal (Fig. 1C). Quantification of human monocyte-derived DCs (moDCs) chemotaxis towards CCL21 in the absence or presence of 0.1–10 µM C21TP (71–111) demonstrated that CCL21-induced chemotaxis was boosted significantly by the C21TP in a dose-dependent manner by up to 25-fold in the presence of the highest peptide concentration tested (10 µM) (Fig. 1C, D). Video presentation illustrating DC chemotaxis towards 10 nM CCL21 in the presence or absence of C21TP is available as supplementary information on CMIs website. This effect is also clearly appreciated when studying the morphology of the migrating DCs (Fig. 1E). In the presence of CCL21 alone, DCs display a somewhat contracted, quiescent form, whereas in the presence of both CCL21 and C21TP (TP71-111) the DCs completely change their morphology to display highly extended dendritic structures extending towards the chemokine source (to the left). C21TP alone did not induce DC chemotaxis (data not shown) suggesting that C21TP renders the DCs more sensitive to CCL21.

### C21TP changes the signaling profile of CCL21

**Fig. 2:**
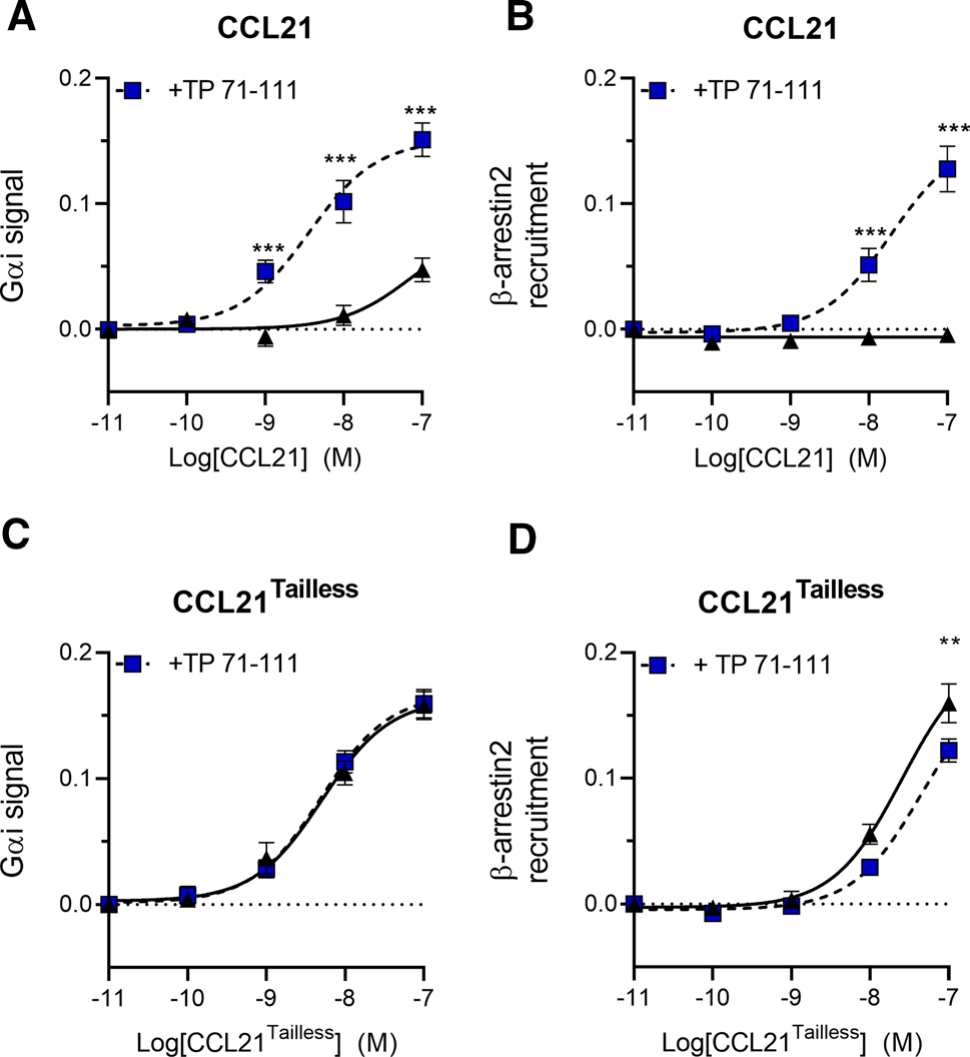
C21TP boosts CCL21 potency in G_αi_ signaling and β-arrestin recruitment. The effect of C21TP (TP71-111) on G_αi_–signaling (**A**, **C**) and β-arrestin recruitment potential (**B**, **D**) in response to CCL21 (**A**, **B**) or CCL21^Tailless^ (CCL21 1–79) (**C**, **D**) was measured using BRET-based assays. C21TP (TP71-111) was added for a final concentration of 10 µM. Black triangles: chemokine alone, blue squares: chemokine in the presence of TP71-111. Statistical significances was determined using two-way ANOVA with Sidak’s multiple comparisons test (*n* = 4). **P < 0.01, ***P < 0.001

After establishing a strong boosting effect of C21TP on DC migration, we focused on more receptor-close signaling pathways, usually elicited by chemokine receptors (G_αi_ signaling and arrestin recruitment). At CCR7, both CCL19 and CCL21 induce G protein signaling, with a higher potency of CCL19. Both chemokines are also able to stimulate arrestin recruitment, although the effect of CCL21 is extremely limited and often undetectable [3, 19]. Using BRET based reporter systems C21TP was evaluated for its influence on CCL21-induced CCR7 activity. Consistent with previous data [4], CCL21 signaling through G_αi_ displayed low potency (Fig. 2A) and β-arrestin recruitment by CCR7 in response to CCL21 alone could not be detected at all (Fig. 2B). Both CCL21 induced G_αi_ signaling and β-arrestin recruitment were boosted extensively in the presence of 10 µM C21TP (TP71-111) (G_αi_ signaling: CCL21 alone pEC_50_ 6.46 versus CCL21 + TP71-111 pEC_50_ 8.43) (β-arrestin recruitment: CCL21 alone undetectable versus CCL21 + TP71-111 pEC_50_ 7.72) (Fig. 2A, B). Activity of CCL21^Tailless^ (CCL21 lacking the C-terminal tail, CCL21 1–79) that as shown previously is more potent than CCL21 [4], was not improved by the presence of free C21TP (TP71-111) (Fig. 2C, D). In fact, in the presence of C21TP the chemokine displayed a small but significant decrease in its potential to induce β-arrestin recruitment.

### Orientation but not length of C21TP is essential for retained boosting

**Fig. 3:**
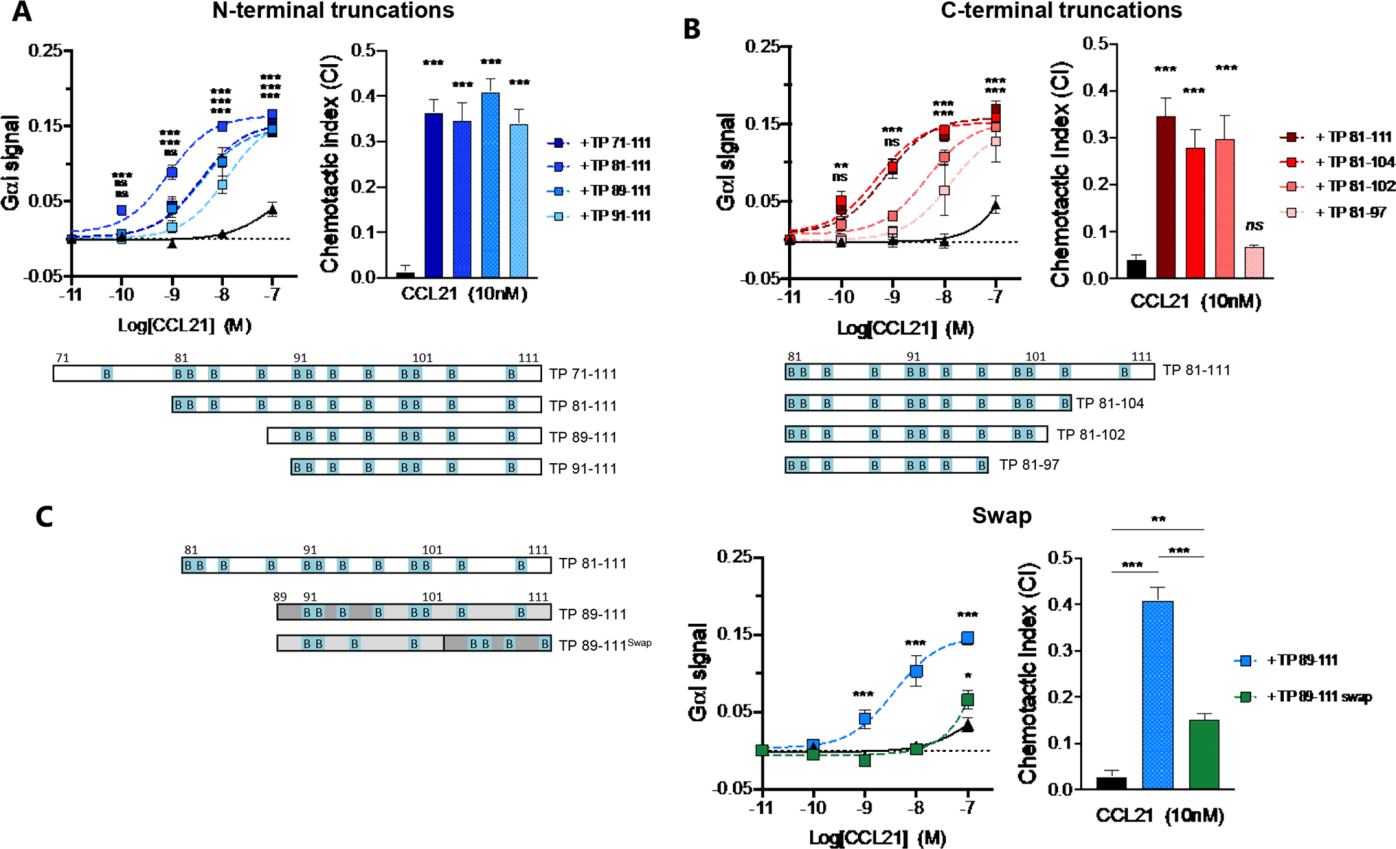
Peptide orientation but not length of C21TP is essential for retained boosting. The length and location of BBx(x)B domains in the various peptides are shown as graphical bars. The location and distribution of positive charged residues (lysines or arginines) are highlighted and identified with *B*. Signaling and migration were tested employing the BRET-based cAMP sensor assay and time-lapse recordings of human moDCs naturally expressing CCR7. C21TP TP71-111 and all peptide variants were added to a final concentration of 10 µM. Peptide effect on DC migration was assessed in the presence of 10 nM CCL21. CCL21 boosting effect of various **A** N-terminal and **B** C-terminal truncated peptides. **C** The boosting ability of C21TP TP89-111 and its swapped variant, TP89-111swap on CCL21 activity. Black symbols and bars: CCL21 alone, various blue, red and green symbols; CCL21 + C21TP variants. Statistical significances between signaling curves were calculated using two-way ANOVA with Dunnett’s multiple comparisons tests, and statistical significances between CI values were calculated by one-way ANOVA with either Dunnett’s (**A**, **B**) or Tukey’s (**C**) correction for multiple test (*n* = 3–8). *P < 0.05, **P < 0.01, ***P < 0.001. *ns* not significant. In **A** and **B** the *P* values for different peptides are grouped; **A** (upper: TP-81–111, middle: TP-71–111 and TP89-111, lower: TP91-111) **B** (upper: TP81-111 and TP81-104, lower: TP81-102 and TP81-97)

Inspired by the strong boosting effect of C21TP on CCL21 activity, we moved on to determine the minimal sequence of C21TP retaining boosting ability. Therefore, a set of different N-terminally and C-terminally truncated versions of the original TP71-111 were designed and tested. First, the naturally occurring peptide generated upon plasmin cleavage TP81-111 was tested and found to boost CCL21-induced chemotaxis at least to the same extent as TP71-111, whereas G_αi_ signaling was boosted even more by TP81-111 (Fig. 3A). Further truncation revealed that C21TP could be reduced N-terminally by 20 aa to TP91-111 while retaining boosting ability (Fig. 3A). C-terminal truncation of the most potent peptide, TP81-111, also revealed conserved boosting effects of peptides down to TP81-102, whereas further C-terminal reduction to TP81-97, reduced signaling via cAMP and blunted chemotaxis boosting ability (Fig. 3B).

Chemokine GAG affinity is conferred by basic motifs that are traditionally described to follow the consensus sequence BBx(x)B, where B refers to basic amino acids and x to any amino acid. To explore if it is the number of GAG binding motifs or the actual sequence orientation that determines the C21TP boosting ability, we interchanged the N-terminal and C-terminal parts of the shortest peptide with retained full boosting activity compared to TP71-111. Thus, TP89-111 and its swap variant TP98-111│89–97 (from now on referred to as TP89-111swap) were tested in parallel for their boosting effect on CCL21 induced G_αi_ signaling and DC chemotaxis. The boosting of CCL21-induced signaling and chemotaxis was severely diminished in the presence of TP89-111swap compared to TP89-111 (Fig. 3C). This finding underscores the importance of peptide sequence orientation for retained boosting effect of CCL21. Surface plasmon resonance analysis revealed that the two C21TP variants, TP89-111 and TP89-111swap, had similar affinity for the GAG heparan sulfate (Kd 2.93 µM vs 2.96 µM) (data not shown) indicating that the boosting capacity towards CCL21 may not be directly linked to interference with GAG binding per se.

### C21TP also potentiates CCL19 induced G_αi_ signaling, β-arrestin recruitment and chemotaxis

**Fig. 4:**
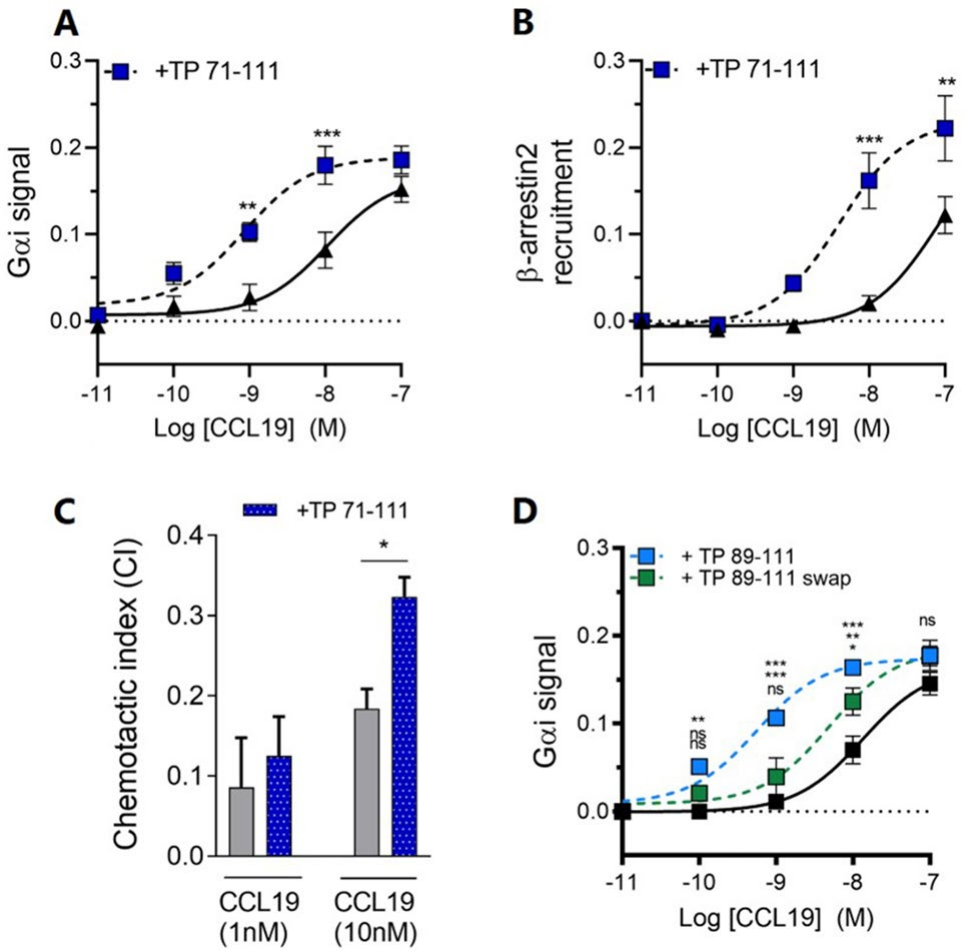
C21TP boosts CCL19 potency in G_αi_ signaling, β-arrestin recruitment and chemotaxis. **A**, **B** The effect of C21TP on CCL19-dependent G_αi_ signaling and β-arrestin recruitment potential was measured using BRET-based assays. TP71-111 was added to a final concentration of 10 µM. Black triangles: chemokine alone, blue squares: chemokine in the presence of TP71-111. Statistical significances was determined using two-way ANOVA with Sidak’s multiple comparisons test (*n* = 4). *P < 0.05, **P < 0.01, *P* < 0.001. **C** The effect of TP71-111 on CCL19-induced chemotaxis measured by time-lapse recordings of human moDCs naturally expressing CCR7. Statistical significance was calculated using an unpaired *t* test (*n* = 3–6). **D** Boosting of CCL19 induced G_ai_ –signaling in the presence of TP89-111 and the swapped variant TP89-111swap. Black symbols; CCL19 alone, blue symbol; CCL19 + TP89-111, green symbol; CCL19 + TP89-111swap. Statistical significance was calculated using two-way ANOVA with Tukey’s correction for multiple test. *P* values are reported as; upper: no peptide vs TP 89–111, middle: TP 89–111 vs TP 89-111swap, lower: no peptide vs TP89-111swap (*n* = 3). *P < 0.05, **P < 0.01, ***P < 0.001. *ns* not significant

To investigate if the strong boosting effect of C21TP observed for CCL21 is ligand dependent, we tested its effect on CCL19 induced signaling in the same pathways. As previously reported, CCL19 alone is an efficient inducer of CCR7 mediated G_αi_ signaling and β-arrestin recruitment (G_αi_ signaling: pEC_50_ 7.94, β-arrestin recruitment: pEC_50_ 7.11) (Fig. 4A, B). Intriguingly, the potency of both G_αi_ signaling and β-arrestin recruitment induced by CCL19 was significantly boosted by the addition of the C21TP (TP71-111) (G_αi_ signaling: CCL19 + TP71-111 pEC_50_ 9.58, β-arrestin recruitment: CCL19 + TP71-111 pEC_50_ 8.39). In line with earlier data, CCL19, but not CCL21, induced DC chemotaxis at a concentration of 10 nM [4]. Still, chemotaxis towards 10 nM CCL19 was significantly increased in the presence of 10 µM C21TP (TP71-111), by approximately twofold (Fig. 4C). As observed for CCL21, the swapped peptide version of TP89-111, TP89-111swap, was highly reduced in its boosting of CCL19 (Fig. 4D).

### C21TP boosting is independent of its GAG binding

**Fig. 5:**
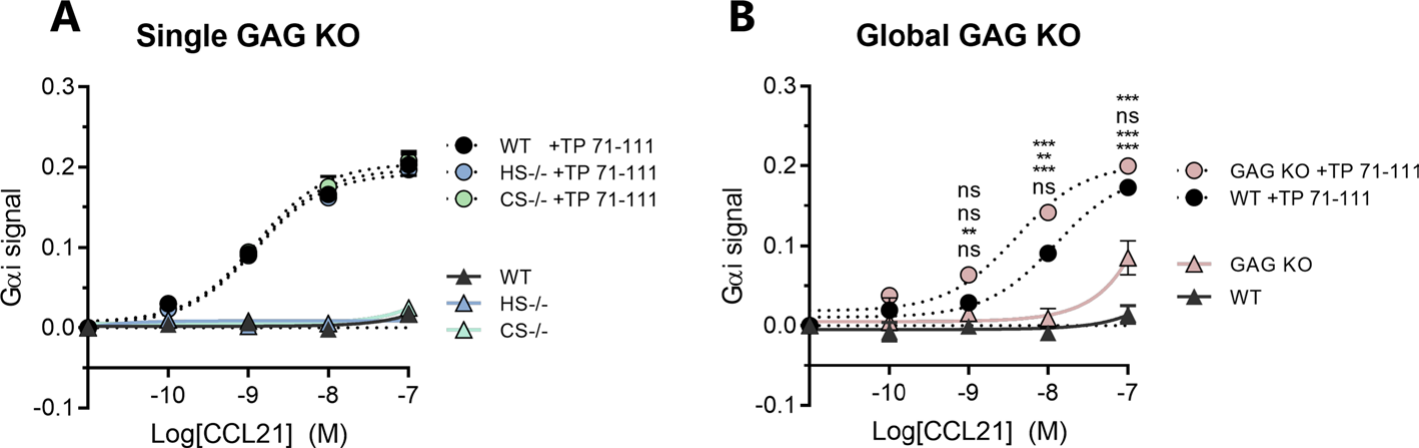
C21TP boosting ability is independent of cell GAG status. **A** Signaling in CHO cells devoid of a single GAG type, either heparan sulphate (HS−/−) or chondroitin sulphate (CS−/−). Cell lines are from the published GAGOme cell library [23] (*n* = 3). **B** Signaling in CHO cells with a complete removal of GAGs [24], called GAG KO. Signaling was quantified using the BRET based cAMP assay. C21TP (TP71-111) was added to a final concentration of 10 µM (*n* = 3). Statistical significance was calculated using two-way ANOVA with Tukey’s correction for multiple test. In **A**, no significant differences are seen between the three different cell line In **B**, *P* values are reported as following starting from the upper value: WT vs WT + TP71-111, WT + TP71-111 vs KO + TP71-111, KO vs KO + TP71-111, WT vs KO. **P < 0.01, ***P < 0.001. *ns* not significant

The potentiation of CCL19 by C21TP was surprising, since the elongated C-term in CCL21 is not mirrored by CCL19, and thus CCL19 does not possess strong GAG binding or adopts an auto-inhibited conformation as suggested for CCL21 [4, 11, 12]. This, together with the fact that C21TP version TP89-111swap was unable to boost either of the two ligands despite its retained GAG affinity, argue against a mechanism relying on GAG chemokine displacement for boosting capacity. To fully exclude that C21TP potentiation of CCL21 is dependent of GAGs and thus not due to the release of a GAG retained chemokine pool, we quantified C21TP boosting effect of CCL21 in cells devoid of GAG synthesis. The C21TP TP71-111 boosting effect was measured in two cell-lines from the recently published GAGOme cell library, genetically manipulated to knock out heparan sulfate expression (EXTL 2/3^−/−^ here referred to as HS−/−) and chondroitin sulfate expression (CSGALNACT 1/2^−/−^ and CHSY1^−/−^ referred to as CS−/−), respectively [23]. The peptide was also tested in a cell-line completely deficient in GAG synthesis due to lack of endogenous B4GalT7 activity [24] (CHO-pgsB 618 referred to as GAG KO). In none of the GAG-deficient cells did we measure any decrease in C21TP ability of CCR7 signaling induced by CCL21 (Fig. 5A). In fact, there was a tendency that boosting by C21TP (TP71-111) was increased in the GAG KO cells (Fig. 5B). Boosting of CCL19 was similarly unaffected by the lack of GAGs (Supplementary Fig. 1).

### BBxB motifs in the C-terminus of chemokines may dictate responsiveness to C21TP boosting

To define what makes a chemokine sensitive to C21TP boosting, we compared the C-terminal parts of the chemokines CCL21, CCL21^Tailless^ and CCL19. Alignment of these chemokines made it clear the CCL19 contains a small tail piece that is very basic in nature, which is not the case for CCL21^Tailless^ (Supplementary Fig. 2A). Another chemokine, CCL20, acts through CCR6 that is expressed by immature DCs and is important for their guidance to inflamed tissues [25]. CCL20 resembles CCL19 with regard to having a BBXB motif positioned at the extreme C-terminus. Signaling induced by CCL20 at CCR6 displays boosting by C21TP (TP71-111) very similar to the boosting observed with CCL19 acting at CCR7 (Supplementary Fig. 2B).

### C21TP boosting relies on *O*-glycosylation in the N-terminus of CCR7

**Fig. 6:**
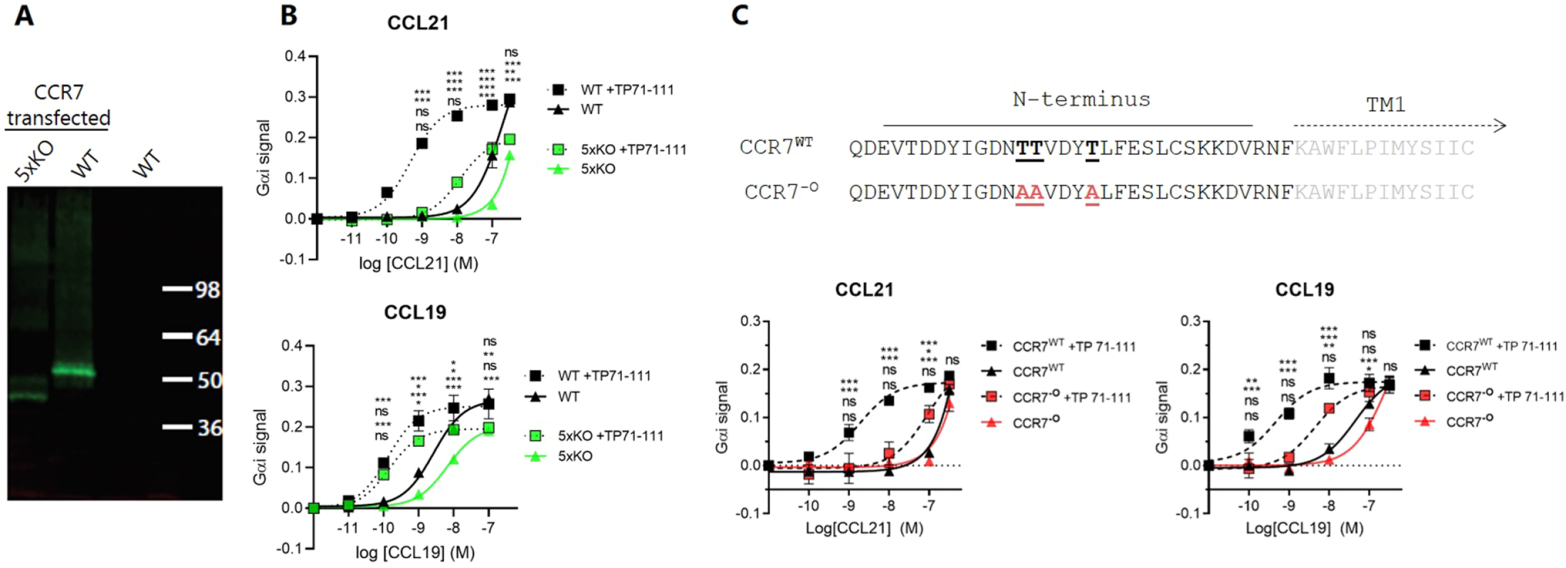
C21TP boosting ability depends on *O*-glycosylation sites in the CCR7 N-terminus. **A** Western blot of CCR7 expressed in WT CHO cells or the 5xKO CHO cell line, incapable of initiating *O*-glycosylation. The cell line is designated 5xKO due to the knock out of five different GalNAc-Ts (GalNAc-T1, 2, 4, 7 and 10). The first two lanes contain cell lysates from cells transfected with YFP-tagged CCR7, while the third contains lysate from untransfected WT cells. **B** CCL21 or CCL19 induced CCR7 G_αi_ –signaling in CHO WT or 5xKO cells quantified using the BRET based cAMP assay. C21TP (TP71-111) was added to a final concentration of 10 µM (*n* = 3). WT alone (black triangle), WT + TP71-111 (black square), 5xKO alone (green triangle), 5xKO + TP71-111 (green square). *P* values are reported as following starting from the upper value: WT vs WT + TP71-111, WT + TP71-111 vs 5xKO + TP71-111, 5xKO vs 5xKO + TP71-111, WT vs 5xKO. **C** G_αi_ –signaling of WT CCR7 or CCR7^−O^ lacking N-terminal *O*-glycosylation sites. The top depict the sequence of WT CCR7 and CCR^−O^. Previously reported [28] N-terminal *O*-glycosylation sites in CCR7, underlined and marked in bold, have been mutated to alanine to prevent the addition of *O*-glycans in CCR7^−O^ (CCR7 T37/38/42A). CCR7 contains a 24-residue long signal peptide cleaved of from the mature protein [51], why the depicted sequence starts at Q25. G_αi_ –signaling was quantified using the BRET based cAMP assay. C21TP (TP71-111) was added to a final concentration of 10 µM (*n* = 3). WT alone (black triangle), WT + TP71-111 (black square), CCR^−O^ alone (red triangle), CCR^−O^ + TP71-111 (red square). *P* values are reported as following starting from the upper value: WT vs WT + TP71-111, WT + TP71-111 vs O- + TP71-111, O- vs O- + TP71-111, WT vs O-. Statistical significance was calculated using two-way ANOVA with Tukey’s correction for multiple test. *P < 0.05, **P < 0.01, ***P < 0.001. *ns* not significant

Since we believed the basic nature of the C21TP to be important for its boosting ability, we examined whethernegatively charged structures other than GAG could influence peptide function. Polysialylation of CCR7 has been reported to unlock the auto-inhibited conformation of CCL21 [13]; however, polysialyltransferases are not ubiquitously expressed and polysialylation has been most thoroughly investigated in the central nervous system. In contrast, every single cell is capable of *N*- and *O*-glycosylation and these structures are usually capped with a single sialic acid [26, 27]. Additionally, Hauser et al. demonstrated that sialylation on CCR7 *N*-glycans affects its functionality [14] and a recent glycoproteomics study identified three specific *O*-glycosylation sites in the CCR7 N-terminus in human CEM T cells [28]. Glycans have also been shown to affect functionality of murine CCR7 [29]. Consequently, we decided to investigate the functional role of CCR7 O-glycosylation in C21TP boosting. To analyze the glycosylation status of CCR7 in our model cell line, we employed a genetically engineered cell line with knockout of five different GalNAc-transferases (designated 5xKO). By western blot, we observed a downwards molecular shift of CCR7, when expressed in CHO 5xKO compared to the wild-type CHO cells (Fig. 6A).

This confirms that CCR7 is O-glycosylated in CHO cells. CCR7 signaling analysis in the CHO 5xKO cells revealed that the lack of glycosylation impaired signaling elicited by CCL21 to a minor extent, whereas C21TP (TP71-111) boosting effect of CCL21 induced signaling was reduced 25-fold from pEC_50_ 9.3 to 7.9 (Fig. 6B). Lack of glycosylation only slightly impaired CCL19 signaling in the absence or presence of C21TP.

Since the 5xKO cells have impairment of all O-glycan structures, we next evaluated the effect of *O*-glycan structures in CCR7 only through construction of a mutant version of CCR7 lacking previously reported extracellular *O*-linked glycosylation sites, CCR7-O (CCR7 T37/38/42A) [28] (Fig. 6C). CHO cells transiently transfected with either CCR7 wild type (WT) or mutated CCR7-O were tested for signaling via G_αi_ (WT and CCR7-O constructs are expressed at similar levels, Supplementary Fig. 3). In contrast to C21TP boosting of signaling via WT CCR7, C21TP (TP71-111) boosting of CCL21 signaling via the glycosylation deficient receptor version was drastically reduced from pEC_50_ 8.7 (CCR7^WT^) to 7.0 (CCR7^−O^) although chemokine activity on its own was almost unaffected by removal of the *O*-linked glycosylations in the CCR7 N-terminus (Fig. 6C). Boosting of CCL19 signaling was also diminished (Fig. 6C). Thus, whereas the *O*-glycosylation of CCR7 does not seem to be critical for the normal interaction between CCR7 and either CCL21 or CCL19, the highly negatively charged *O*-glycan structures are important for C21TP boosting of ligand-induced CCR7 activity. As the 5xKO cells have a different origin than the cells used to assess CCR7-O, and since the glycan synthesis KO affects the entire cell, these signaling results cannot be compared directly, but together the data obtained here suggest that *O*-glycosylation of CCR7 is central for the boosting potential of C21TP. We also tested the signaling induced by CCL21 through CCR7 lacking previously reported extracellular *N*-linked glycosylation sites [14], CCR7-N (CCR7 N36/292A). Removal of extracellular *N*-glycosylation sites had no effect on basal signaling induced by CCL21 nor C21TP (TP71-111) boosting of this signaling (Supplementary Fig. 4).

### C21TP binds to the *O*-glycosylated CCR7 N-terminus

**Fig. 7:**
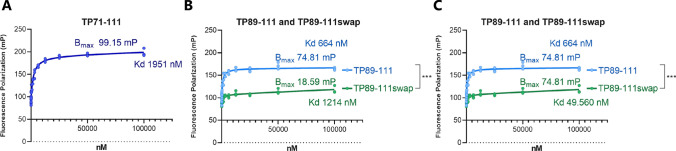
C21TP binds to CCR7 glycosylated N-terminus. Fluorescence polarization assay measuring binding between *O*-glycosylated CCR7 receptor N-terminus and C21TP. The glycosylated CCR7 N-terminus encompasses aa 25–57 of CCR7 (without signal peptide aa 1–24) and an *O*-glycosylation at position T38, as this is the position that was singlehandedly most important for the boosting effect (data not shown). The peptide was N-terminally tagged with Fluorescein (FAM) and C-terminally tagged with biotin. A fixed concentration of 250 nM CCR7 N-terminal peptide was used with the addition of various C21TP in a twofold dilution series for final concentrations ranging from 12.21 nM to 100 µM (*n* = 3). **A** Binding of the original full length C21TP TP71-111. **B** Binding of the truncated TP89-111 and swapped variant TP89-111swap with individual fitting of binding curves. **C** Binding of the truncated TP89-111 and swapped variant TP89-111swap with a fixed *B*_max_, the *B*_max_ determined for TP89-111 in *B*. *B*_max_ and *K*_d_ values were determined by fitting hyperbolic binding curves. Statistical significance were calculated using two-way anova, ***P < 0.001

In order to test if C21TP binds to the glycosylated CCR7 N-terminus, we designed a peptide encompassing aa 25–57 of CCR7 (without signal peptide aa 1–24), that was *O*-glycosylated at position T38, as this is the position that was singlehandedly most important for the boosting effect (data not shown). The *O*-glycosylated CCR7 N-terminus was N-terminally tagged with Fluorescein (FAM) and C-terminally tagged with biotin. Using fluorescence polarization assay, we measured binding of the C21TP versions TP71-111, TP89-111 and TP89-111swap to the *O*-glycosylated CCR7 N-terminus. We found that the fluorescence polarization of the *O*-glycosylated CCR7 N-terminal peptide increased in a dose dependent manner upon addition of the original C21TP TP71-111 yielding a hyperbolic binding curve and a dissociation constant (*K*_d_) of ~ 1900 nM. (Fig. 7A). The truncated C21TP version TP89-111 also bound to the *O*-glycosylated CCR7 N-terminus with a hyperbolic binding curvature, whereas the TP89-111swap displayed unspecific binding properties (Fig. 7B). The lower *B*_max_ of TP89-111 compared to TP71-111 reflects the smaller size of the truncated C21TP version and was thus expected, whereas the differences in *B*_max_ for TP89-111 and the TP89-111swap version, which have the same size, reflects the lack of binding between the *O*-glycosylated CCR7 N-terminal peptide and TP89-111swap. The *K*_d_ of TP89-111swap is arbitrary as it reflects the very low *B*_max_ of the swapped C21TP peptide. Constraining the *B*_max_ of the swapped peptide to that of TP89-111 yields a *K*_d_ of 50 µM (Fig. 7C), implying that the swapped version, TP89-111swap in fact does not show any binding affinity for the O-glycosylated CCR7 N-terminal peptide.

## Discussion

In the current study, we show that the basic C-terminal fragment of CCL21, C21TP (TP71-111 and variants), potentiates the activity of CCL21 at the chemokine receptor CCR7. The C21TP peptide extensively boosts the chemotaxis potential, G_αi_ and arrestin recruitment activity of CCL21, and surprisingly also boosts the activity of the chemokine CCL19, but not the truncated version of CCL21, CCL21^Tailless^, although all three induce activation of CCR7.

GAG retention may shield ligands from productive interactions with their receptors. Thus at low chemokine concentrations, high-affinity GAG-binding chemokines are sequestered in interactions with GAGs instead of interacting with their cognate receptor, a phenomenon that can be counteracted by high chemokine concentrations of either the same or other GAG binding chemokines [18]. GAGs are highly sulfated and chemokine affinity for GAGs relies especially on the stereochemistry and spacing of negatively charged sulfate groups within a specific GAG subtype [30]. CCL21 is a strong GAG binder by virtue of its basic C-terminus. The inherent GAG binding nature of the CCL21 C-terminus, together with our observations that C21TP did not affect CCL21^Tailless^ potency, initially led us to speculate that the boosting of CCL21 by excess free C21TP was due to release of a GAG-retained CCL21 chemokine reservoir. However, this hypothesis turned out not to be true. First, the signaling induced by CCL19, which has a low affinity for GAGs, was also boosted by C21TP (Fig. 4). Second, neither normal ligand signaling behavior nor the boosting effect of C21TP was affected by alterations in the cell GAG repertoire or even complete GAG removal (Fig. 5). Last, the swapped peptide of C21TP TP98-111swap that retained the high GAG affinity of the original TP89-111 peptide was severely weakened in its ability to boost G_αi_ activity and chemotaxis potential of CCL21 (Fig. 3C).

In the current study we have used Collagen I in the 3D chemotaxis assays. Collagen I has low affinity for CCL21 [31]. Matrigel on the other hand binds CCL21 well. As CCL21 is immobilized in vivo [20], to evaluate the full effect of C21TP on dendritic cell migration, experiments using Matrigel would need to be conducted. Such assay could reveal if C21TP affect the interaction between CCR7 and immobilized CCL21 where the basic tail is participating in surface tethering and potentially unable to engage in steric hindrance of CCR7. Again, since boosting of in vitro signaling occurred in both presence and absence of GAGs in CHO cells in vitro, boosting of chemotaxis in Matrigel is expected to occur as well.

Another phenomenon that could explain the C21TP boosting ability would be that the peptide was able to relieve the auto-inhibition of CCL21. The peptide could potentially occupy the site on CCL21 normally contacted by the attached basic C-terminus during the auto-inhibition process, in this way preventing the formation of the locked CCL21 version. In this way the peptide could participate in unfolding of CCL21, similar to the process that is predicted to take place in the presence of polysialic acid residues [13]. The boosting effect of C21TP on CCL19 could also be based on a direct chemokine core domain interaction with the peptide possibly forcing CCL19 into an even more favorable conformation for interaction with CCR7. The fact that the peptide orientation is key for upholding boosting ability indicates that the peptide interacts in a somewhat ordered manner with the entities that it contacts to cause boosting. On the other hand, as the CCL21 self-association is weak and only detectable at mM concentrations [22], a direct interaction of the C-terminus of one CCL21 molecule with the core domain of another is not likely. This contradicts the hypothesis that the free tail peptide binds to the core domain of CCL21, and such mechanism, therefore, is unlikely to be the determining factor for the boosting ability of C21TP.

Polysialyation of CCR7 in DCs is a known mechanism regulating CCL21 activity in vivo [13]. In cells incapable of polysialylation, sulfated tyrosines and other receptor modifications of acidic nature are known to play a regulatory function as well. In CCR5, sialylated *O*-glycans and sulfated tyrosines in the N-terminus have been shown to be important for both CCL3 (MIP-1α) and CCL4 (MIP-1β) binding and signaling [32]. Similarly, sulfated tyrosines in CCR7 seem to play a role in its interaction with CCL21, where N-terminal peptides lacking these modifications displayed reduced affinities for CCL21 [16]. Three specific *O*-glycosylation sites have been detected in CCR7 by tandem mass spectometry in human CEM T cells [28]. Additionally, these sites are predicted to be *O*-glycosylated by the NetOglyc 4.0 prediction algoritm [33] and finally we were able to confirm that CCR7 carries *O*-glycosylation in CHO cells, shown by the shift in mass when expressed in wt and GalNAc-T KO cells (5xKO) (Fig. 6A). We, therefore, speculated that *O*-glycosylated residues on CCR7 could be of importance for CCL21 interaction with CCR7 and that the C21TP boosting could guide CCL21 to a more favorable interaction through shielding of negative sialic acid residues in the CCR7 N-terminus allowing for a freer docking mode of CCL21 despite its basic C-terminus.

We found that removal of the reported *O*-glycosylation sites [28] in the CCR7 N-terminus abrogated C21TP boosting ability, without affecting basal CCR7 activation via CCL19 and CCL21. This demonstrates that *O*-glycosylations of CCR7 are not important for the chemokine-mediated activity as such in CHO cells, but the boosting conveyed by the free C21TP somehow involves interaction of the peptide with glycan structures in the CCR7 N-terminus. Removal of the glycosylation sites did not completely abolish peptide boosting of CCR7, and it is possible that tyrosine sulfation of the CCR7 N-terminus also influences boosting by the tail peptide. An analysis of a combination of glycosylation and tyrosine sulfation sites is required to completely dissect the molecular basis of the boosting effect. Removal of previously reported extracellular N-glycosylation sites in CCR7 [14] did not affect basal signaling induced by CCL21 nor did it affect C21TP boosting of CCL21 signaling that remained as with WT CCR7 (Supplementary Fig. 3). Our data are in contrast to data reported by Hauser et al. [14] that observed an increase in CCL21 induced signaling upon removal of these sites. Hauser et al. used 300-19 cells and measured Ca^2+^ mobilization and directed migration, whereas we used CHO cells and measured G_αi_ signaling. Moreover, the CCR7 constructs used by Hauser et al. carried a HA-tag (HA-tag: YPYDVPDYA) and a double strep tag (Strep tag: WSHPQFEK) in the N-terminus, whereas we used an un-tagged CCR7 construct. The tags contain both negative and positively charged amino acids and when positioned in the N-terminus of CCR7 could possibly influence the behavior of the N-terminus and its interaction partners. Further studies remain to determine whether the difference between our results and Hauser’s relies on the difference in receptor constructs and or cell-lines.

To our surprise, C21TP was also able to boost CCL19 but not CCL21^Tailless^. An explanation for this could be that although CCL19 displays a very weak GAG affinity [11, 12], it may also perform some charge–charge interactions with the N-terminal glycans in CCR7. The C-terminal region of CCL19 contains a cluster of basic residues, not mirrored in CCL21^Tailless^ (Supplementary Fig. 2A), and some of these residues form a BBxB motif. Although CCL19 is a strong inducer of CCR7 activity, this basic cluster may also constrain CCL19 in its engagement with CCR7, explaining why we do observe some potentiation of CCL19 by C21TP. This hypothesis is backed up by the fact that C21TP also boosts CCL20 signaling via CCR6, since CCL20 also contains a BBxB motif in the C-terminus (Supplementary Fig. 2B). Therefore, the C21TP boosting seems to be somewhat directed towards chemokines that have basic C-terminus conforming to a classical BBxB motif, although further studies are needed to confirm this. Interestingly, CCR6 has two predicted N-terminal *O*-glycosylation sites [34].

Many studies suggest that most chemokine receptors form constitutive or ligand-induced homo- and/or heterodimers and that these dimers modulate receptor activities [35, 36]. Homodimerization of CCR7 was recently shown to increase CCL19 binding and enhance CCL19 induced migration of T cells [36]. Thus, there is a possibility that C21TP may induce CCR7 dimerization affecting ligand-induced receptor activation, but the fact that signaling induced by CCL21^Tailless^ is not boosted by C21TP contradicts this. It could be argued that CCR7 in its dimerized state is only activated by CCL19 and CCL21 but not CCL21^Tailless^, but then we would expect signaling induced by CCL21^Tailless^ to be counteracted by C21TP, as most receptors would be sequestered in a dimerized state, which is not the case. Another scenario is that the peptide induces ligand-dependent dimerization of CCR7 through a mechanism that involves glycans in the CCR7 N-terminus, which is interesting and something that could be addressed in a future study.

In the absence of peptide, CCL19 and CCL21 contact CCR7 in different manners [4]. The interaction of CCL21 depends on residues in the top of TM-3, -4 and -5 that are not important for CCL19 interaction with CCR7. The binding of these two chemokines induce different structural changes in CCR7 to disrupt old and create new hydrogen bonds [6]. CCL21 binding to CCR7 is believed to involve movements within the majority of helical domains (TM2, -3, -4, -5, -6, and -7), whereas the changes induced by CCL19 are restricted to regions of TM3, -5, and -6 [37]. The conformational changes in CCR7 induced by CCL19 lead to β–arrestin recruitment, whereas the changes induced by CCL21 only do so to a minor extent [3, 6]. We have previously reported that the chemokine N-terminus is not a determining factor for the biased signaling observed with CCL19 and CCL21, with only CCL19 inducing CCR7 β-arrestin recruitment [10]. It might be the overall approach instead, by which the chemokines dock into CCR7, which determines the conformational changes and thus elicitation of signaling pathways. One major point of bias in signaling between CCL19 and CCL21 is found in the β-arrestin refractory CCR7 state induced by CCL21. The change in CCL21-induced receptor signaling from a β-arrestin refractory to a β-arrestin recruiting state in the presence of C21TP indicates that CCR7 adopts a different conformation when activated by CCL21 in the absence versus presence of tail-peptide. Such changes in activation mode are likely coupled to different docking modes of the ligand in the absence versus presence of tail peptide [6]. Structural and dynamic studies of the beta-2 adrenergic receptor in the presence or absence of ligand imply that the receptor occupies intermediate conformational states, which accommodates the structure of the ligand to facilitate its docking. [38]. Recently, the structure of CCR7 bound to an allosteric antagonist, Cmp2105, was solved. Cmp2105 was shown to fix CCR7 in an inactive conformation through high affinity binding to the cytosolic side [39]. The binding site of Cmp2105 overlaps with the G-protein binding site antagonizing the structural changes required to accommodate receptor activation, but at the same time Cmp2105 outcompetes CCL19 binding to CCR7, indicating that the fixed inactive state does not allow ligand docking. C21TP on the other hand could possibly allow CCR7 to adopt a conformation more fitted for effective ligand docking. The fact that CCL19 is more potent than CCL21 in inducing activation of CCR7 as measured by activation of most downstream signaling pathways could lead one to believe that CCL19 has a higher affinity for CCR7. This is not the case, as earlier studies report similar binding affinities for CCL19 and CCL21 towards CCR7. Thus in a study by Yoshida et al. [40], CCL19 and CCL21 displayed similar binding affinities in L.1.2 cells in a homologues competition binding assay. CCL21 was in the same study shown to have a sixfold lower binding affinity in primary T cells, but this was estimated employing a heterologous competition binding assay that only estimates apparent affinity. Based on these data, we find it unlikely that C21TP boosting effect is due to effects relating to binding affinities, but rather C21TP affects docking mode and accompanying helical rearrangements that improve signaling via G_αi_ and beta-arrestin, a notion that is also supported by the fact that differences in signaling observed between CCL19 and CCL21 relates to differential docking modes [4, 6].

**Fig. 8:**
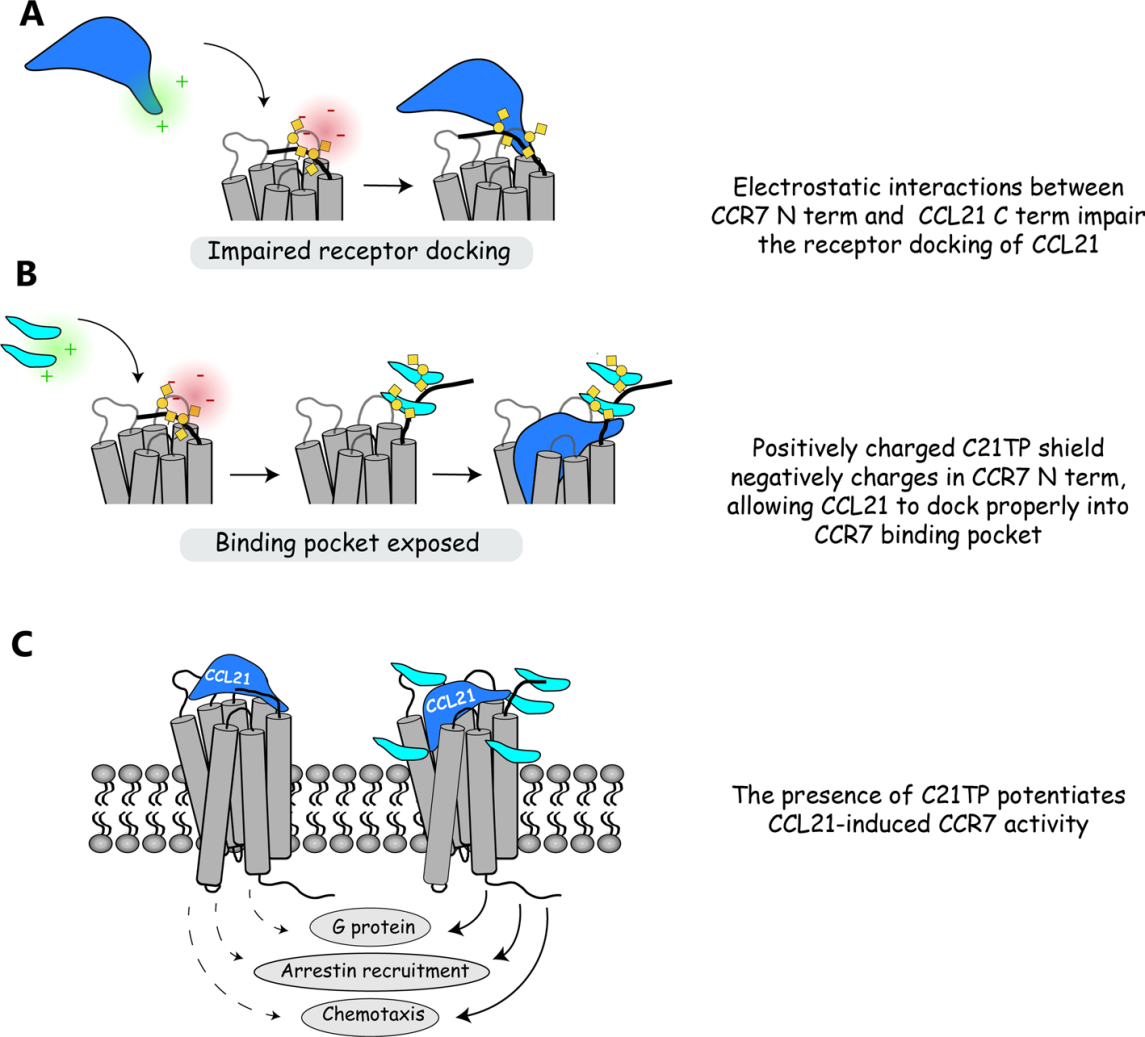
Proposed model for C21TP boosting action on CCL21 induced CCR7 signaling. **A** In the absence of C21TP CCL21 is a weak stimulator of CCR7 activity. We propose that positively charged residues in CCL21 interacts with negatively charged entities in CCR7, presumably glycan structures in the N-terminus, restraining CCL21 in a conformation incompatible with proper docking into the binding pocket. **B** In the presence of excess C21TP (light blue fragment) the positively charged peptide(s) interacts with the negative entities in CCR7 N-term. When C21TP shields the negative charges, CCL21 is able to dock properly into the CCR7 binding pocket and thus leading to a strong receptor activation. **C** The weak CCL21 induced CCR7 activity in G protein signaling, arrestin recruitment and chemotaxis assays is potentiated in the presence of excess C21TP

We cannot exclude that C21TP boosts CCL21 activity by relieving the auto inhibitory conformation described for CCL21 [13]. However, we found that CCR7 *O*-glycans are important for boosting of CCL21 and CCL19 and that C21TP binds directly to the *O*-glycosylated CCR7 N-terminus (Fig. 7). Here, we were also able to show a direct relationship between O-glycosylated CCR7 N-terminus binding and boosting ability with the two TP89-111 and TP89-111swap version. Based on these findings, we propose a mechanism, which either independently or in synergy with the relieved autoinhibition of CCL21 boosts CCR7 activity (Fig. 8). We propose that interactions between the CCR7 N-terminus and CCL21 restrain the chemokine’s ability to dock into the binding pocket of CCR7 (Fig. 8A). Electrostatic interactions between the positively charged C21TP and negatively charged CCR7 N-terminus glycan structures re-organize the conformation of CCR7 creating a more accessible binding pocket facilitating the docking of CCL21 (Fig. 8B) and CCL19. Both CCL21 and CCL19 contain clusters of basic residues in their C-terminal region not mirrored in CCL21^Tailless^ (Supplementary Fig. 2A) that could explain why the shielding of negatively charged glycan structures in CCR7 by C21TP would improve signaling of CCL21 (Fig. 8C) and CCL19 but not CCL21^Tailless^.

CCL19 and CCL21 induce different conformational changes in CCR7 on their own [6], with CCL19 inducing a receptor state that is more active than CCL21. C21TP seems to be able to correct this and thus it is perceivable that C21TP causes CCL21 to engage with CCR7 in a way that causes similar helical rearrangements as those induced upon CCL19 receptor engagement.

What role endogenous-produced C21TP plays under normal physiological conditions and whether it affects the activity of lymphatic or LN expressed and positioned CCL21 is not clear. As C21TP, due to its basic nature, is extremely sticky, unspecific binding to plastic containers and cell components (negatively charged) during an in vitro setup is expected to be high. Due to its small size and since it is added either before or at the same time as CCL21, comparably more C21TP will be lost to unspecific binding compared to CCL21. Therefore, it is likely that in site in vivo generation of C21TP, generated through cleavage of CCL21 at the DC surface, need not be that much in excess to have a local effect on the interaction of un-cleaved CCL21 with CCR7.

CCL21 expressed by the small lymphatic capillaries is of importance for re-localization of peripheral DCs to the LNs, guiding both trans-endothelial migration and intra-lymphatic crawling and CCL21 blockage has been shown to block DC migration from lymph capillaries to collecting ducts [41, 42]. Lymphatic capillaries are 15–60 µm in diameter [43] and DCs are likely fill up the lumen of these during intra-lymphatic crawling. From earlier studies, we established that DCs when chemotaxing within narrow channels towards a CCL21 gradient, where no fluid exchange is possible between front and rear of the cell, significantly increase their chemotaxis speed [44], possibly due to the buildup of a steeper gradient in front of the migrating DCs. As cleavage of CCL21 by DC secreted proteases occurs locally, at the DC surface, to generate CCL21^Tailless^ and C21TP, the concentration of both molecules at the DC surface could be a lot higher than that of CCL21. Local boosting of full length CCL21 by C21TP at the front of each migrating DC, together with CCL21^Tailless^, generated through the action of DC secreted proteases, together could possibly be of great importance for DC chemotaxis speed. As ACKR4 leads to internalization of CCL21 and CCL21^Tailless^ [45], but not C21TP, this could contribute to the disproportionate accumulation of C21TP, inside the lymphatic vessels and in the LN, that could possibly affect the activity of CCL21 released from the lymphatic endothelium.

Overall, our data indicate the possible use of C21TP in immunotherapeutic treatment, where reactivation of the immune system is an advantage (e.g. cancer treatment). Importantly, dendritic cells (DCs) are the most potent antigen presenting cells and increasingly appreciated as key to turning cold tumors hot, a prerequisite for success of most other immune oncology (IO) therapies, including CAR-T [46, 47]. Current DC vaccine therapies suffer from overall low efficacy due to amongst other things, poor lymph node homing of the injected DCs [48]; C21TP may be able to boost homing of injected DCs and thus vaccine efficacy by potentiating local CCL21 reservoirs inside lymphatic vessels. Thorough in vivo testing is required to substantiate this theory, investigations that are beyond the scope of this study.

## Conclusion

In the current work, we show that the low potency of CCL21 can be boosted by the C-terminal domain of CCL21 as a free tail peptide, C21TP. The potentiation of CCL21 is not mediated by the release of a GAG retained chemokine reservoir as first hypothesized, but through a mechanism that depends on C21TP interaction with N-terminally positioned *O*-glycans in CCR7. We speculate that this interaction facilitates docking of ligands with positively charged C-termini, causing a more favorable interaction with CCR7 with following helical rearrangements that support both G_αi_ signaling and β-arrestin recruitment.

## Materials and methods

### Materials

X-vivo 15 medium was from Lonza (Basel, Switzerland). CaCl_2_, MgCl_2_, glucose, HEPES, Human AB serum, Na_2_HCO_3_ (7.5%), MEM (10X), FBS, Penicillin/Streptomycin, glutamine, PGE2, Forskolin, formaldehyde and Fluoromount were from Sigma (St. Louis, MO, USA). IL-4, GM-CSF, TNF-α, IL-1β, and IL-6, were from Peprotech (Rocky Hill, NJ, USA). DMEM, RPMI, PBS, Trypsin and HBSS were from Thermo Scientific (Waltham, MA, USA). Lymphoprep was from STEMCELL Technologies (Vancouver, Canada). PureCol Bovine Collagen I suspension was from Advanced Biomatrix (Carlsbad, CA, USA). CCL19, CCL21 and anti-CCL21 were from R&D Systems (Minneopolis, MN, USA). CCL21^Tailless^ was from Brian Volkmans laboratory [22]. Coelenterazine was from Nanoligth (Pinetop, AZ, USA). Ibidi 3D chemotaxis slides were from Ibidi (Martinsried, Germany). C21TP variants were from Caslo (Lyngby, Denmark). Odyssey blocking buffer and goat anti-mouse antibody IRdye 800cw were from LI-COR Biosciences (Lincoln, NE, USA). Tween-20 was from Merck (Kenilworth, NJ, USA).

O-glycosylation deficient CCR7 was generated from WT through Quick change PCR using the following primers:

CCR7 forward primer: T37/38/42A:

CGATTACATCGGAGACAACGCCGCAGTGGACTACGCTTTGTTCGAGTCTTTGTGC

CCR7 reverse primer: T37/38/42A:

GCACAAAGACTCGAACAAAGTGTAGTCCACTGTGGTGTTGTCTCCGATGTAATCG

The glycosylated CCR7 N-terminal peptide was custom synthesized by Sussex Research Laboratories Inc. (Ontario, Canada). The peptide encompass 5-FAM-QDEVTDDYIGDNTT*VDYTLFESLCSKKDVRNF-K(Biotin)-OH, where * denotes glycosylation at threonine T38 and FAM = Fluorescein. The glycosylation is *O*-α-(Neu5Acα(2–3)Galβ(1–3)GalNac).

### Dendritic cell (DC) preparation

Buffy coats were obtained from Rigshospitalet Copenhagen, as anonymous material, with written informed consent from the donors. The local ethics committee at Faculty of Health and Medical Sciences at the University of Copenhagen (Research Ethics Committee for Sund and Science, University of Copenhagen) found the project exempt from approval. DCs were prepared from human peripheral blood mononuclear cells (PBMC) isolated from buffy coats by centrifugation on a Lymphoprep gradient as previously described [49]. Briefly, monocytes were isolated by plastic adherence of PBMC. Adhered monocytes were subsequently cultured and differentiated into immature DCs by incubation with IL-4 (250 U/ml) and GM-CSF (1000 U/ml) for 6 days, followed by activation into mature DCs by incubation with IL-6 (1000 U/ml), IL-1β (1000 U/ml), TNF-α (1000 U/ml), and PGE2 (1 µg/ml) for an additional 2 days in the same medium.

### Cell culturing

Human DCs were cultured in X-vivo 15 medium with 2% human AB serum and glutamine. CHO-K1 pgsB-618 ATCC^®^ CRL2241™ [24] and CHO-K1 cells were grown in RPMI with 10% FBS and Penicillin/Streptomycin. HS^−/−^ (CHO EXTL 2/3 −/−), CS^−/−^ (CSGALNACT 1/2^−/−^ CHSY1^−/−^), 5xKO (GalNAc-T1, 2, 4, 7 and 10 −/−) and WT CHO suspension cells were grown in suspension in 1:1 of EX-Cell CD CHO fusion media and Balanced CD CHO growth A medium supplemented with 2% glutamine and Penicillin/Streptomycin. Cells were kept in a humidified incubator at 37 °C, 5% CO_2_. For stable cell-lines the passage number did not exceed 40.

### Three-dimensional (3D) chemotaxis

Chemotaxis assays were conducted as previously described [4]. Briefly, mature human moDCs were left to acclimatize in medium for 30 min at room temperature (RT) upon defrosting, before assay start. DCs were seeded in Bovine Collagen I mixture prepared by mixing 10 µl Na_2_HCO_3_ (7.5%), 20 µl MEM (10×), 150 µl PureCol and 90 µl DCs dissolved in X-vivo 15 medium (2 × 10^6^ cells/ml). After incubation for 45 min in a humidified incubator at 37 °C (5% CO_2_), the source and sink reservoirs were filled according to the manufacturer’s instructions and chemotaxis was tracked in a time-lapse microscope with a humidified temperature controlled stage incubator for 12 h at a 2 min interval. Cell migration (approximately 20–40 cells per viewing field) was tracked using a commercial tracking program (Autozell) and subsequently analyzed to get a population-based chemotactic index (CI) value (MATLAB). CI is a measure of net translocation distance to the source relative to total distance traveled and was thus calculated as the ratio of the distance traveled in the direction of the gradient over the total distance traveled and therefore is a conservative measure of the directedness of cell migration.

### Bioluminescence resonance energy transfer (BRET) cAMP and β-arrestin assay

For cAMP assays, adherent CHO cells were transfected using lipofectamine while suspension cells were transfected using the FectoPro method. Cells were seeded in a 6-well plate, either 500,000 (adherent cells) or 200,000 (suspension cells) cells/well. The next day the cells were transiently transfected with vectors encoding the human CCR7 and CAMYEL sensor (cAMP sensor using YFP-Epac-RLuc) [50] in a 1:5 ratio using lipofectamine (6 µl/well) or FectoPro (4 µl/well). Lipofectamine transfections were terminated by changing to 2 ml fresh cultivation medium after 5 h, while Fectopro transfection was terminated after 3 h by adding 2.5 ml fresh cultivation medium and 2 µl FectoPro Boost reagent per well. The cells were incubated under standard cultivation conditions overnight. For β-arrestin recruitment assays, cells were transfected with CCR7 and vectors expressing a Renilla luciferase arrestin3 fusion protein and membrane-SH3-citrine protein. The cells were then resuspended in PBS w/glucose and seeded in 96-well white iso plates (~ 25,000 cells/well). Coelenterazine (bioluminescence substrate) was added to a final conc. of 5 µM. After 10 min, cells were stimulated with varying ligand concentrations for a total of 40 min. Forskolin was added to each well 5 min after ligand addition to reach a final conc. of 5 µM. The plates were kept in the dark at all times. Emission signal from Rluc and eYFP was measured using the Envision machine at 530 and 480 nm and the BRET signal determined as the ratio between (eYFP/Rluc) [50].

### Western blot to confirm the *O*-glycosylation status of CCR7 in CHO cells

Cells were seeded in a 6-well plate and transfected with 1 μg of C-terminal M1-tagged CCR7. 24 h post transfection, cells were harvested and lysed in RIPA buffer. Samples were run on a 4–15% Criterion TGX gel and blotted to a PVDF membrane using the Biorad trans blot turbo system. Membranes were blocked for 45 min in blocking buffer and incubated with anti-M1 antibody in 1:10,000 dilution ON at 4C in 50% blocking buffer and PBS with Ca and Mg. The following day, the membrane was washed three times in PBS with 0.05% Tween-20 and incubated with anti-mouse antibody dilution 1:5000 (IRdye 800cw goat anti-mouse from LI-COR). Bands were then visualized on LI-COR imager and the picture was cropped and labeled using Adobe illustrator.

### Fluorescence polarization (FP) assay

The carboxyfluorescein (FAM) labeled peptide representing the CCR7 N-terminus glycosylated at T38 functioned as the probe for quantifying the interaction between the N-terminus and the C21TP versions; TP71-111, TP89-111 and TP89-111swap. The probe was diluted in FP buffer (1:3, PBS: MilliQ) and 10 µl was added to a final concentration of 250 nM per well in a black 384-well plate (Corning). The C21 tail peptides (2 mM) were diluted using a twofold dilution series. 30 µl of the tail peptides was added to each well to final concentrations ranging from 12.21 nM to 100 µM. Blanks containing 10 µl probe and 30 µl FP buffer were included. The plate was spun down at 1000 rpm for 1 min and covered with adhesive film. The FP signal was measured on a Flex station using the SoftMax Pro software. Each experiment was conducted in triplicate. The binding curves were generated by plotting the FP signal, expressed as millipolarization units (mP), against C21TP concentration. The maximum specific binding (*B*_max_) and the dissociation constant (*K*_d_) values were calculated using GraphPad Prism 9.

## Supplementary Information

Below is the link to the electronic supplementary material.

Supplementary file1 C21TP boosting of CCL19 is independent of cell GAG status. (A) Signaling in CHO cells devoid of a single GAG type, either heparan sulphate (HS-/-) or chondroitin sulphate (CS-/-). Cells lines are from the published GAGOme cell library [23] (n=3). (B) Signaling in CHO cells with a complete removal of GAGs [24], called GAG KO. Signaling was quantified using the BRET based cAMP assay. C21TP (TP71-111) was added to a final concentration of 10 µM (n=3). Statistical significance was calculated using two-way ANOVA with Tukey’s correction for multiple test. In A, no significant differences are seen between the three different cell lines. In B, P-values are reported as following starting from the upper value: WT vs WT + TP TP71-111, WT +TP TP71-111 vs KO + TP TP71-111, KO vs KO + TP TP71-111, WT vs KO. *P<0.05, **P <0.01, ***P <0.001, ns, not significant (PNG 724 KB)

Supplementary file2 C21TP also boosts signaling of the DC expressed chemokine receptor CCR6. A) Alignment of CCL19, CCL19 and CCL21Tailless. CCL21 contains multiple BBxB motifs in its extended C-terminus, CCL19 also has a short basic tail with a BBxB motif, whereas CCL21Tailless lacks basic amino acids in its C-terminal. B) CCL20-induced CCR6 Gαi –signaling in CHO cells quantified using the BRET Based cAMP assay. C21TP (TP71-111) was added to a final concentration of 10 µM (n=3). CCL20 alone (black triangles), CCL20 +TP71-111 (blue square). Statistical significance was calculated using two-way ANOVA with Sidek’s correction for multiple test. *P<0.05, **P <0.01, ***P <0.001, ns, not significant (PNG 878 KB)

Supplementary file3 CCR7 WT and CCR7-O are expressed to the same level in CHO cells transfected with the receptor constructs. The surface level of WT CCR7 and CCR7-O was quantified by flow analysis of transfected cells. The surface expression of WT CCR7 and CCR7-O are comparable at both receptor construct concentrations tested. Mock transfection: grey, WT CCR7 Green and CCR7-O yellow (PNG 1132 KB)

Supplementary file4 C21TP boosting ability is not dependent on N-glycosylation sites. CCL21-induced CCR7 Gαi –signaling in the absence of previously reported important CCR7 N-glycosylation sites N36 and N292 [14]. Signaling was measured in CHO cells quantified using the BRET based cAMP assay. C21TP (TP71-111) was added to a final concentration of 10 µM (n=3). CCR7WT + TP71-111 (black square), CCR7WT alone (black triangle), CCR7-N+ TP71-111 (blue square), CCR7-N alone (blue triangle). Upper P-values shows the statistical difference between CCR7WT and CCR7-N in the presence of TP71-111, lower P-values show the statistical difference in the absence of peptide. Statistical significance was calculated using two-way ANOVA with Tukey’s correction for multiple test. *P<0.05, **P <0.01, ***P<0.001 (PNG 328 KB)

Supplementary file5 (AVI 7676 KB)

Supplementary file6 (AVI 7797 KB)

## Data Availability

The datasets generated during and/or analysed during the current study are available from the corresponding author on reasonable request.
