# Supplementary figures and images for: The C-terminal peptide of CCL21 drastically augments CCL21 activity through the dendritic cell lymph node homing receptor CCR7 by interaction with the receptor N-terminus
Source: Cell Mol Life Sci. 2021 Sep 29;78(21-22):6963–78. doi: 10.1007/s00018-021-03930-7 (PMC8558179; doi:10.1007/s00018-021-03930-7)

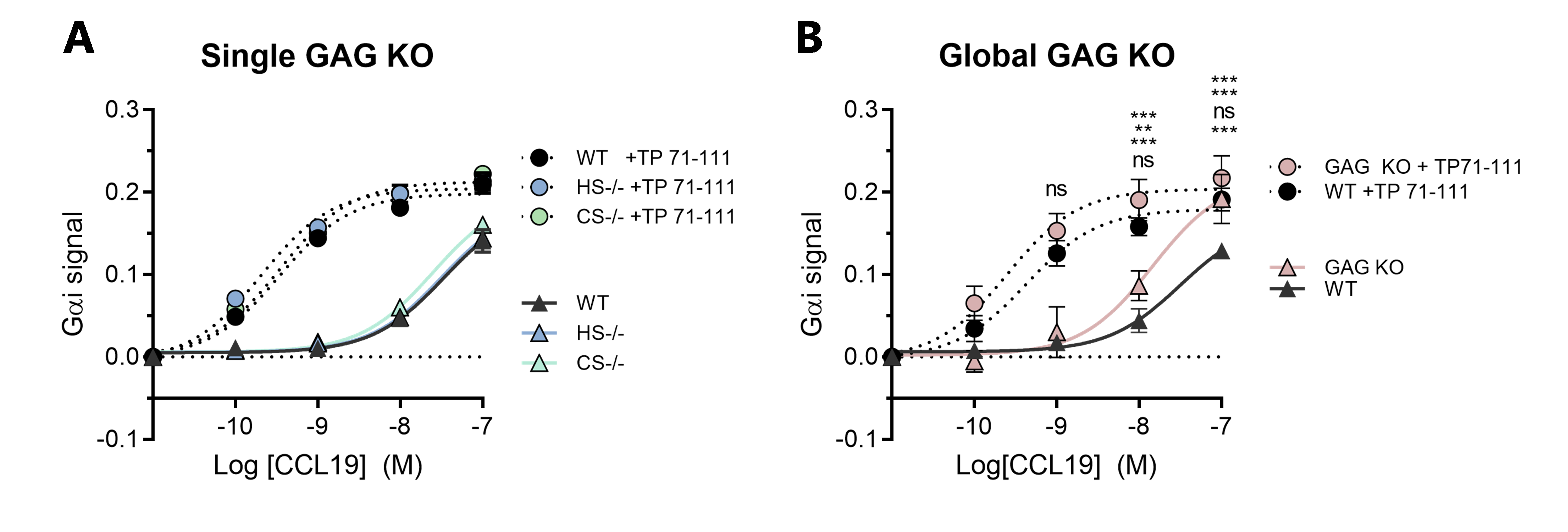

Supplement: Supplementary file 1 — Supplementary file1 C21TP boosting of CCL19 is independent of cell GAG status. (A) Signaling in CHO cells devoid of a single GAG type, either heparan sulphate (HS-/-) or chondroitin sulphate (CS-/-). Cells lines are from the published GAGOme cell library [23] (n=3). (B) Signaling in CHO cells with a complete removal of GAGs [24], called GAG KO. Signaling was quantified using the BRET based cAMP assay. C21TP (TP71-111) was added to a final concentration of 10 µM (n=3). Statistical significance was calculated using two-way ANOVA with Tukey’s correction for multiple test. In A, no significant differences are seen between the three different cell lines. In B, P-values are reported as following starting from the upper value: WT vs WT + TP TP71-111, WT +TP TP71-111 vs KO + TP TP71-111, KO vs KO + TP TP71-111, WT vs KO. *P<0.05, **P <0.01, ***P <0.001, ns, not significant (PNG 724 KB) [file 18_2021_3930_MOESM1_ESM.png]

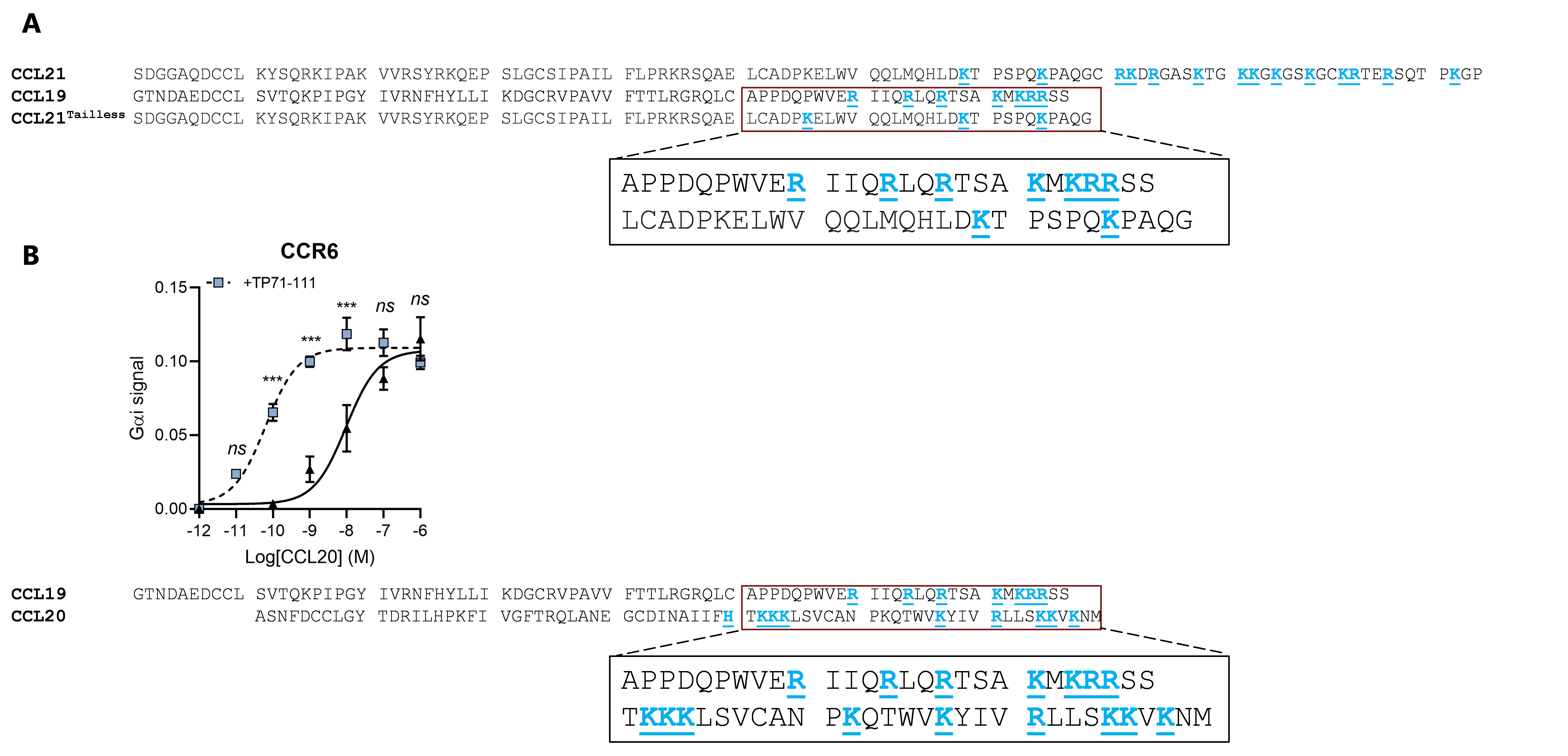

Supplement: Supplementary file 2 — Supplementary file2 C21TP also boosts signaling of the DC expressed chemokine receptor CCR6. A) Alignment of CCL19, CCL19 and CCL21Tailless. CCL21 contains multiple BBxB motifs in its extended C-terminus, CCL19 also has a short basic tail with a BBxB motif, whereas CCL21Tailless lacks basic amino acids in its C-terminal. B) CCL20-induced CCR6 Gαi –signaling in CHO cells quantified using the BRET Based cAMP assay. C21TP (TP71-111) was added to a final concentration of 10 µM (n=3). CCL20 alone (black triangles), CCL20 +TP71-111 (blue square). Statistical significance was calculated using two-way ANOVA with Sidek’s correction for multiple test. *P<0.05, **P <0.01, ***P <0.001, ns, not significant (PNG 878 KB) [file 18_2021_3930_MOESM2_ESM.png]

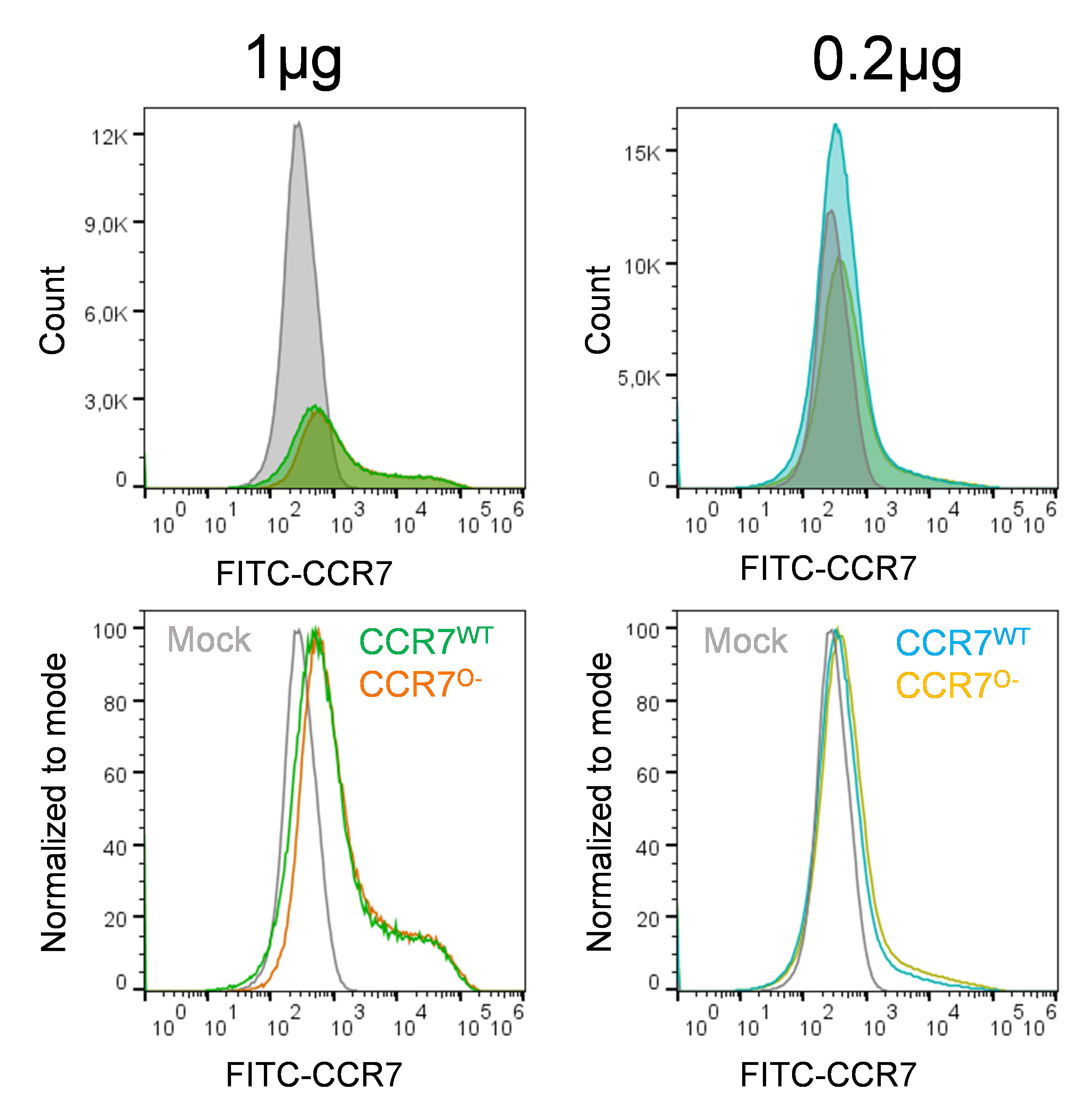

Supplement: Supplementary file 3 — Supplementary file3 CCR7 WT and CCR7-O are expressed to the same level in CHO cells transfected with the receptor constructs. The surface level of WT CCR7 and CCR7-O was quantified by flow analysis of transfected cells. The surface expression of WT CCR7 and CCR7-O are comparable at both receptor construct concentrations tested. Mock transfection: grey, WT CCR7 Green and CCR7-O yellow (PNG 1132 KB) [file 18_2021_3930_MOESM3_ESM.png]

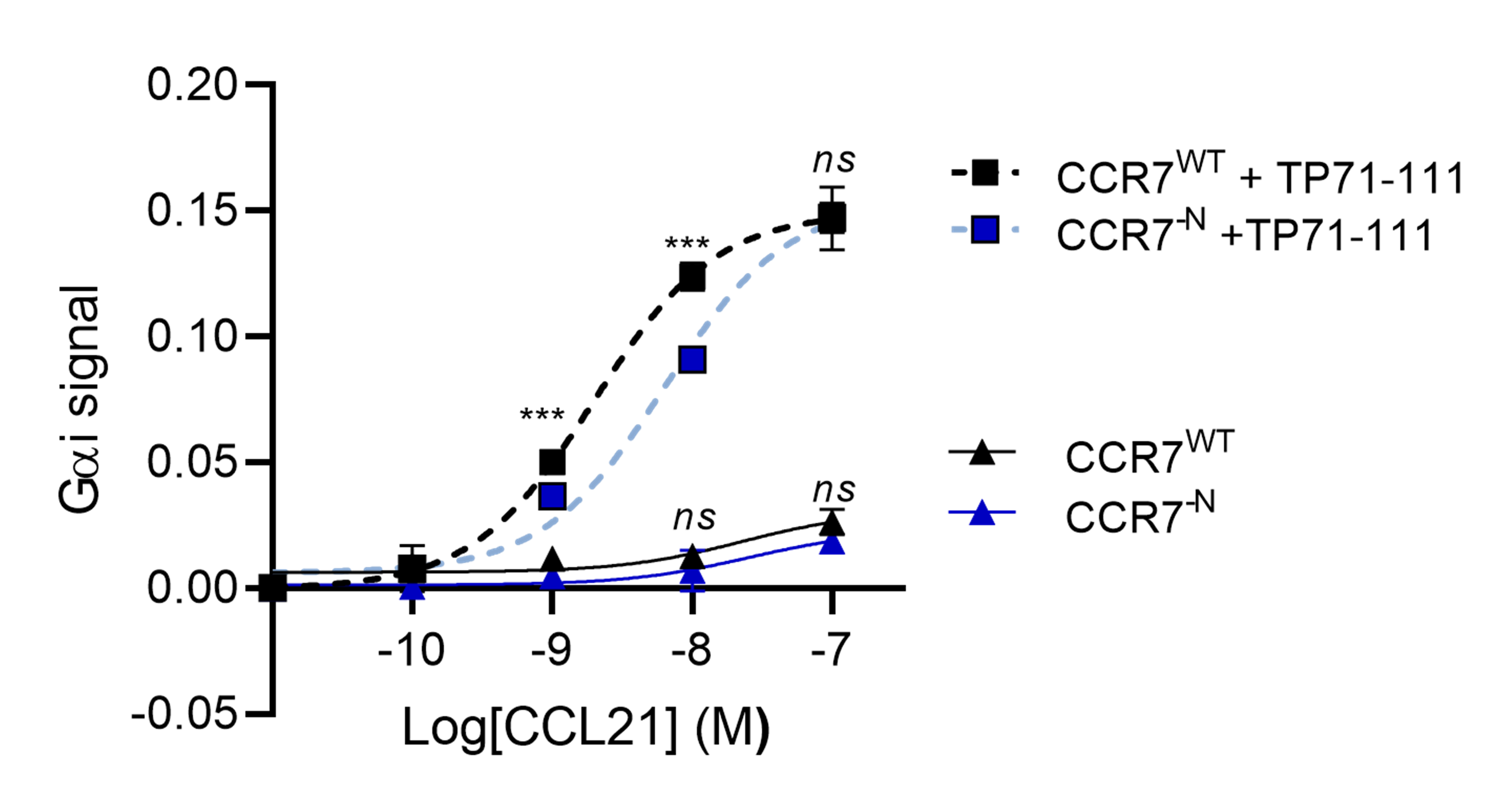

Supplement: Supplementary file 4 — Supplementary file4 C21TP boosting ability is not dependent on N-glycosylation sites. CCL21-induced CCR7 Gαi –signaling in the absence of previously reported important CCR7 N-glycosylation sites N36 and N292 [14]. Signaling was measured in CHO cells quantified using the BRET based cAMP assay. C21TP (TP71-111) was added to a final concentration of 10 µM (n=3). CCR7WT + TP71-111 (black square), CCR7WT alone (black triangle), CCR7-N+ TP71-111 (blue square), CCR7-N alone (blue triangle). Upper P-values shows the statistical difference between CCR7WT and CCR7-N in the presence of TP71-111, lower P-values show the statistical difference in the absence of peptide. Statistical significance was calculated using two-way ANOVA with Tukey’s correction for multiple test. *P<0.05, **P <0.01, ***P<0.001 (PNG 328 KB) [file 18_2021_3930_MOESM4_ESM.png]
